# Evaluation of inactivation of bovine coronavirus by low-level radiofrequency irradiation

**DOI:** 10.1038/s41598-023-36887-7

**Published:** 2023-06-16

**Authors:** Jody C. Cantu, Joseph W. Butterworth, Kevin S. Mylacraine, Bennett L. Ibey, Bryan M. Gamboa, Leland R. Johnson, Robert J. Thomas, Jason A. Payne, William P. Roach, Ibtissam Echchgadda

**Affiliations:** 1 General Dynamics Information Technology, JBSA Fort Sam Houston, TX USA; 2 Air Force Research Laboratory, Radio Frequency Bioeffects Branch, Bioeffects Division, JBSA Fort Sam Houston, TX USA; 3 Air Force Research Laboratory, Bioeffects Division, JBSA Fort Sam Houston, TX USA; 4 Air Force Office of Scientific Research, Air Force Research Laboratory, Arlington, VA USA

**Keywords:** Biochemistry, SARS-CoV-2

## Abstract

Inactivation of influenza A virus by radiofrequency (RF) energy exposure at levels near Institute of Electrical and Electronics Engineers (IEEE) safety thresholds has been reported. The authors hypothesized that this inactivation was through a structure-resonant energy transfer mechanism. If this hypothesis is confirmed, such a technology could be used to prevent transmission of virus in occupied public spaces where RF irradiation of surfaces could be performed at scale. The present study aims to both replicate and expand the previous work by investigating the neutralization of bovine coronavirus (BCoV), a surrogate of SARS-CoV-2, by RF radiation in 6–12 GHz range. Results showed an appreciable reduction in BCoV infectivity (up to 77%) due to RF exposure to certain frequencies, but failed to generate enough reduction to be considered clinically significant.

## Introduction

Sanitization of virus-contaminated spaces is essential to slow the spread of disease^1^. This need was made especially evident during the global COVID-19 pandemic where a coronavirus, SARS-CoV-2 spread quickly in part due to its ability to maintain viability for hours in contaminated areas^2,3^. Current technologies to clean infected spaces include chemicals or ultraviolet treatments^4,5^. Although efficient, these methods require direct contact or line-of-sight with the contaminated area and can be harmful to humans, which limits their deployment in occupied public spaces such as grocery stores and restaurants. Therefore, it is essential to identify technologies that can inhibit viral subsistence safely when employed in such spaces.

A prospective technology capable of wide area viral decontamination is electromagnetic (EM) radiation-based systems in the radiofrequency (RF) range. RF fields are non-ionizing and have the ability to transmit through a range of materials^6^. Previous studies have shown virus neutralization following RF exposure by thermal denaturation (i.e. heat)^7–9^. Specifically, early studies of a retrovirus (Rous sarcoma virus, RSV) showed exposure to pulsed RF (3 GHz, 100 V/cm E-field, 600 ns pulse duration, 500 Hz repetition rate) neutralized RSV under conditions that generated heat^9^. Furthermore, more than 90% inactivation of aerosolized MS2 bacteriophage was shown following exposure to 2.45 GHz (700 W applied power, 900 mW/cm^2^ power density) by inflicting damage to the viral surface visible under electron microscopic examination^8^. However, the power density of the exposure was high enough to generate heat in the virus suspension. Additionally, 2.45 GHz has been shown to neutralize suspensions of hepatitis C virus and human immunodeficiency virus (> 90% neutralization) when exposures induced > 60 °C heating for 3 min^7^. While these studies showed promising evidence that RF heating can induce neutralization of several viruses, the exposure conditions tested in these studies would not be amenable to the creation of a deployable RF technology in occupied spaces, as they generated fields well above IEEE safety standards^10^.

**Figure 1:**
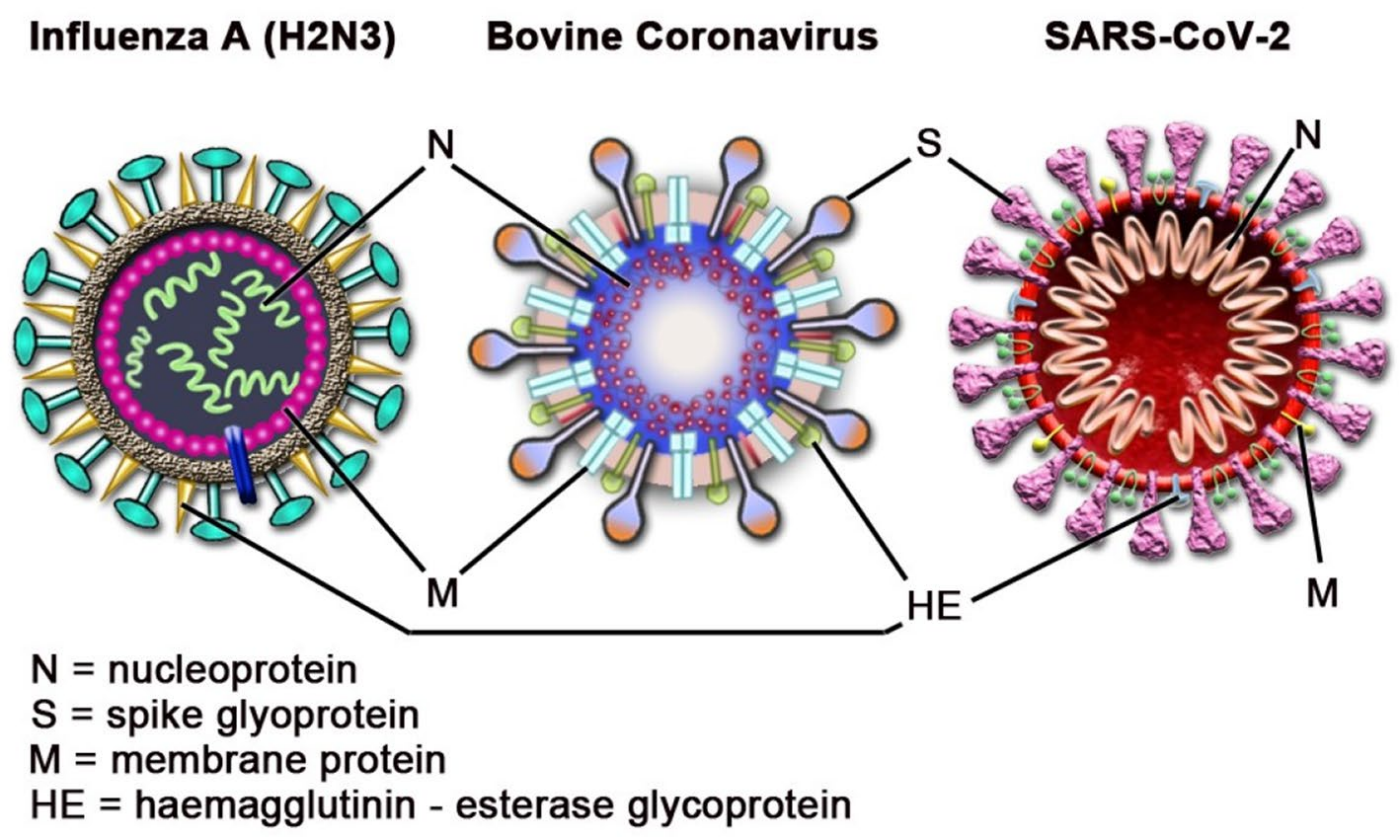
Comparison of BCoV composition to previously studied viruses.

Recent research has reported that RF fields can neutralize viruses under exposure conditions without significant bulk heating of viral samples^11,12^. Specifically, a focusing reflectarray antenna was used to expose a suspension of influenza A type H3N2 virus to a range of continuous wave (CW) RF frequencies (6–18 GHz) at a low power density (1 W applied power, 27 mW/cm^2^ maximum power density, 15 min duration)^11^. The results of this study showed peak virus neutralization at 8 GHz (93%), which the authors defined as the microwave resonant absorption (MRA) frequency of H3N2^11^. A similar effect was also reported by Yang et al., in a study that exposed H3N2 to RF fields (in the range of 6–12 GHz) to excite confined acoustic vibrations (CAVs) inside the virus via a MRA phenomenon^12^. The authors proposed a structure-resonant energy transfer (SRET) mechanism inducing CAVs within the virus by the RF field resulting in physical fracturing of the virus particles^12^. Recently, it has been shown through modeling and experimentation that EM fields can also impact on spike protein confirmation, which is critical for host cell attachment and infection^13,14^. In light of these results, we suggest a similar mechanism could be applicable to the neutralization of coronaviruses, as H3N2 is structurally similar to coronaviruses (Fig. 1)^12^. Specifically, both are enveloped viruses consisting of glycoproteins embedded within a lipid membrane surrounding a ribonucleic acid (RNA) core. Both virions are similar in size, existing as 80–120 nm spherical particles^15–19^. Based on these similarities, we investigated the neutralization of bovine coronavirus (BCoV), a surrogate for SARS-CoV-2, by exposure to RF fields from 6–12 GHz, using an identical exposure setup to that described by Yang et al.^12^ We performed a comprehensive dosimetric analysis of the CW system to enable comparison of any biological trends observed to RF dose. To assess viral infectivity, we performed cytopathic effects (CPE) analysis on HRT-18G host cell cultures infected with BCoV.

## Materials and methods

### HRT-18G host cell culture and bovine coronavirus propagation

Homo sapiens ileocecal colorectal adenocarcinoma cells (HRT-18G, ATCC No. CRL-11663) were obtained from ATCC (Manassas, Virginia). HRT-18G cells were maintained in Dulbecco’s Modified Eagle’s Medium (DMEM) supplemented with 5% fetal bovine serum (FBS) and 100 U/mL penicillin/streptomycin at 37 °C with 5% CO_2_ in air. The media and its components were purchased from ATCC (Manassas, Virginia).

BCoV (NR-445, Mebus) was acquired from BEI Resources (Manassas, Virginia). All experiments containing BCoV were conducted in Eagle’s Minimum Essential Medium (EMEM, ATCC) supplemented with 100 U/mL penicillin/streptomycin. BCoV was propagated to create a virus stock via passage in HRT-18G cells. Specifically, HRT-18G cells were grown to confluent monolayers in 6-well plates. Prior to infection, cell monolayers were rinsed twice with serum-free media (SFM) and inoculated with 200 µL of BCoV BEI stock. Virus inoculated cells were incubated for 1 h (37 °C, 5% CO_2_, 95% relative humidity (RH)) and 1.8 mL of EMEM containing 2% FBS was added to each well. The cells were then incubated for 6 days (37 °C, 5% CO_2_, 95% RH) to allow infection, as evidenced by the appearance of CPE, described in more detail below. This process was repeated twice with reinfection in successively larger cell culture vessels to create a large volume of BCoV stock for experiments. BCoV stock titer was determined and small volume aliquots were prepared and frozen at − 80 °C until needed for experimentation. Before RF exposure, BCoV samples, depending on exposure configuration, were either placed at 1 mL total volume in 1 × 1 × 4 cm plastic cuvettes (Cat#14-377-010, Fisher Scientific) for suspension (solution) samples or were spotted on 25 × 75 mm glass slides (coupon) (Cat#22-037-246, Fisher Scientific) and allowed to dry under the laminar flow hood at room temperature for surface samples.

### Heat controls

**Figure 2:**
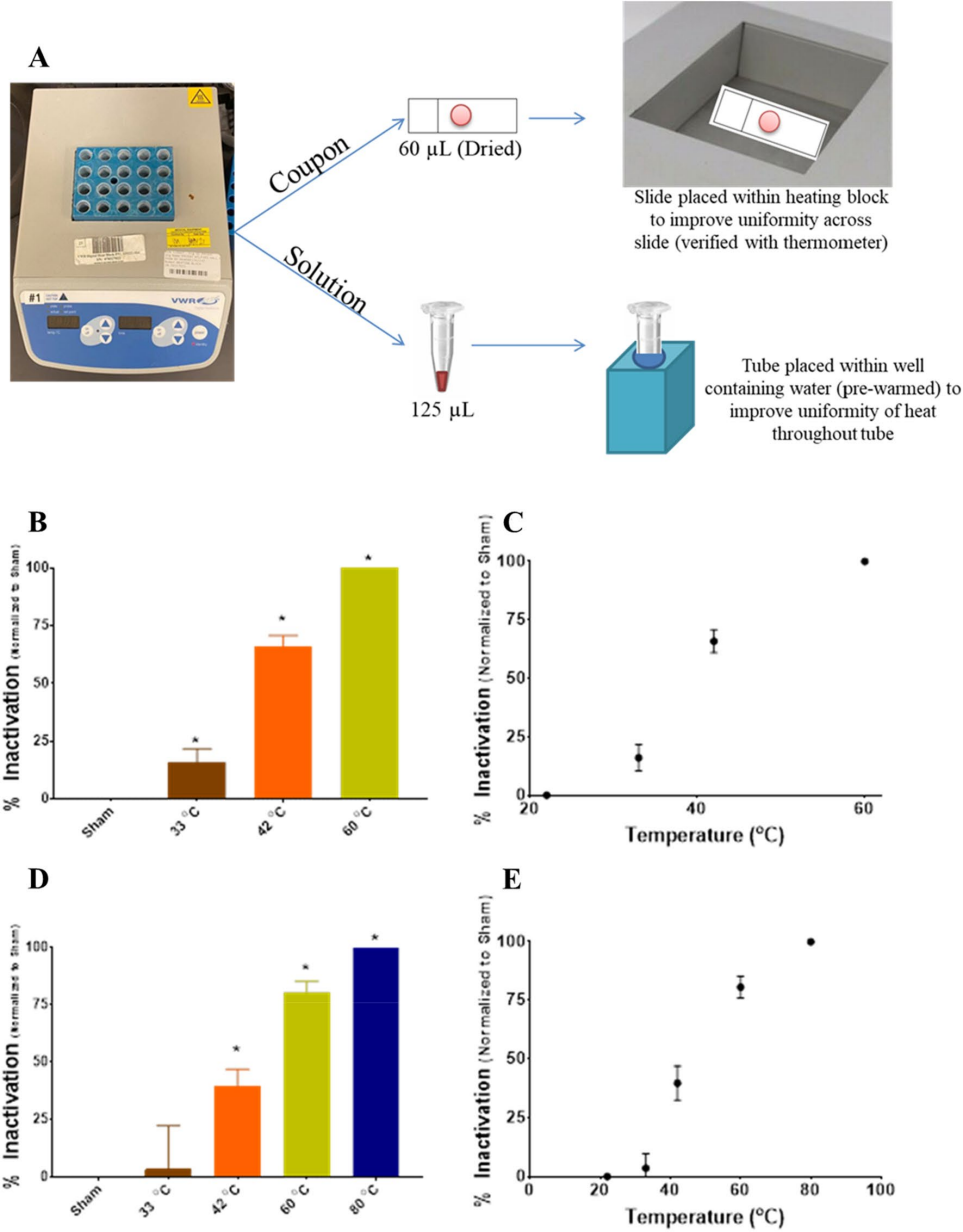
Infectivity of BCoV in solution and in surface after exposure to heat. The experimental setup is shown in panel (**A**). Viral infectivity was assessed by CPE evaluation. Viral titers were determined upon titration on HRT-18G cells, as described in the methods, evaluated at 6 days post inoculation, and normalized to sham. (**B**,**C**) % Inactivation (i.e., data normalized to non-exposed virus sham) following incubation of solutions of BCoV at specific temperatures for 15 min duration plotted in columns or as a function of temperature. (**D**,**E**) % Inactivation following incubation of BCoV dried on glass slides at specific temperatures for 15 min duration plotted in columns or as a function of temperature. Data are expressed as mean values ± S.E.M. of at least three independent experiments (n = 3). Statistically significant differences are noted by an asterisk, which represents p-value < 0.05.

Heating of samples was achieved using a Digital Heat Block (CAT#12621-084, VWR Incorporated, Radnor, PA) (Fig. 2A). The heat block was set to the desired temperature and allowed to equilibrate for 15 min prior to sample exposure. For each surface sample, 60 µL of BCoV stock was spotted on a glass slide and allowed to dry completely. The slide was placed within the heat block to provide the most direct and stable heating of BCoV on surface. After heating at the desired temperature for 15 min (22 °C (control), 33 °C, 42 °C, 60 °C, or 80 °C), virus was eluted from each slide in 120 µL of SFM. For each solution sample, 125 µL of BCoV stock was placed in a 0.5 mL microcentrifuge tube prior to placement in the heat block. To provide uniform heating of the samples, the individual wells of the heat block were filled with water, so that the tubes were floating in an equilibrated solution instead of air. After heating at the desired temperature for 15 min (22 °C (control), 33 °C, 42 °C, or 60 °C), the heat-treated virus samples were then used to infect the HRT-18G cells, and CPE and virus titer was evaluated.

### RF exposure

**Figure 3:**
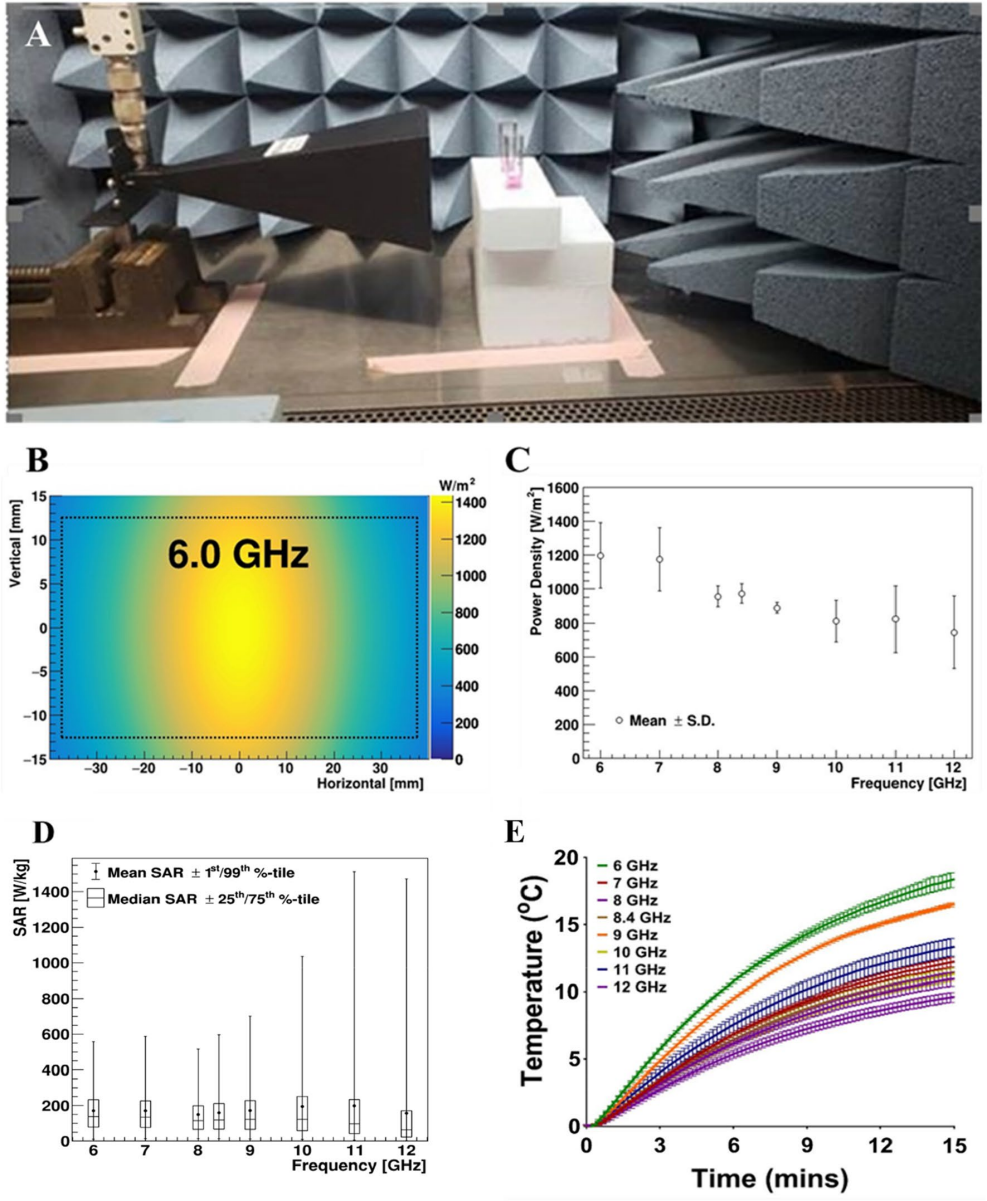
Characterization of the cuvette exposure system. (**A**) Photograph displays the experimental setup for exposure of BCoV in cuvettes. (**B**) Representative numerical simulation of the area of 6.0 GHz exposures using XFdtd^®^ simulation software. Simulations were similar for all tested frequencies. (**C**) The power density was quantified based on the XFdtd^®^ simulation for each free-space exposure and are reported as mean ± standard deviation for each frequency. (**D**) Candle plot of SAR voxel values based on the XFdtd^®^ simulation for each exposure frequency, where the mean, median, 1st, 25th, 75th, and 99th percentile SAR values are depicted. (**E**) Temperature profiles from the cuvette exposures, as measured by a fiber-optic temperature sensor during the 15-min duration. The data are expressed as change in temperature (ΔT) from beginning to end of exposure (mean values ± S.E.M).

A CW field was generated using a 0–12 GHz Signal Generator (SG12000, DS Instruments, Gardnerville, NV), amplified by a 40-W solid state GaN power amplifier (SS18G-40A, EMC Shop, Roseville, CA) and radiated from a horn antenna (ELECTRO-METRICS, EM-6969). This experimental setup was chosen as it mirrored that used by Yang et al.^12^ The aperture size of the antenna was 9.8 × 7.1 cm. To avoid damage to the amplifier caused by back reflection, a bidirectional coupler (Model 3022, L3Harris Narda-MITEQ) was added in line with coaxial source cable before entering the horn antenna. The RF system was placed within a P2 class flow hood. The antenna was directed toward the side for suspension sample exposures (Fig. 3A) or the bottom for surface sample exposure (Fig. 6A) of the flow hood. The RF was normally incident on either the acrylic cuvettes or glass slides at a distance of 5 cm from the exit of horn antenna. To avoid large reflection from the metal hood surface, broadband pyramidal absorbers (EHP-12PCL, Ets-Lindgrin), specified at 45–50 dB attenuation from 4 to 2 GHz, were placed within the chamber (Figs. 3A and 6A). Samples were exposed for 15 min at the specified microwave frequencies / power. Virus samples in cuvettes and glass slides were exposed at integer frequency increments between 6 and 12 GHz. The sham (control) samples consisted of virus on coupon (i.e. spotted on 25 × 75 mm glass slides, further detailed in the methods section) or in suspension that were mock exposed with no RF illumination. Based upon the resulting datasets, we performed a refinement study to investigate whether a resonant peak of inactivation could be reached, along with a series of tests at varied power levels or exposure durations to observe dose–response trends by altering the source power and the total time of exposure.

### Dosimetry

We performed three-dimensional (3D) computational modeling of the empirical exposure conditions to characterize the spatial distribution of incident fields at the viral sample location. Simulations were performed within a commercial Finite-Difference Time-Domain (FDTD) simulation package (XFdtd^®^, Remcom^®^, State College, PA), where a Computer-aided Design of the EM6969 horn was used to propagate microwave energy in a free-space environment. We scaled the simulation runs such that the propagated power matched the forward power measurements acquired during the experiments. For each of the eight frequencies (6, 7, 8, 8.4, 9, 10, 11, and 12 GHz), we recorded the simulated power density distribution in a plane 5 cm away from the horn.

For the exposure configurations with the acrylic cuvettes, numerical dosimetry was performed to determine the specific absorption rate (SAR) in the exposed solution for each voxel,

1 $$\mathrm{SAR}\left(r\right)=\frac{\sigma \left(r\right){\left|E\left(r\right)\right|}^{2}}{\rho \left(r\right)},$$

where *r* is the voxel index, σ is the electric conductivity, *E* is the root-mean-square of the steady-state electric field, and ρ is the mass density. The simulation space surrounding the horn and the two solution-filled cuvettes had a global grid edge resolution = 1.5 mm and an inner region surrounding the cuvettes and a grid edge resolution = 0.05 mm. To minimize numerical artifacts, a gradient in edge resolutions was used to transition between the two regions. Each solution volume consisted of 200 × 200 × 200 voxels. The dielectric properties of the solution and cuvette, provided in Table 1, were assumed to be biological saline^20^ and polymethylmethacrylate^21^.

**Table 1:** Dielectric properties for numerical dosimetry.

Frequency (GHz)	Solution ε_r_	Solution σ (S/m)	Cuvette ε_r_	Cuvette σ (S/m)
6.0	69.7374	8.9468	2.626	0.003857
7.0	67.278	11.25	2.626	0.0045
8.0	64.682	13.6968	2.626	0.005142
8.4	63.618	14.702	2.626	0.0054
9.0	62.006	16.23	2.626	0.005785
10.0	59.301	18.796	2.626	0.006428
11.0	56.61	21.36	2.626	0.007071
12.0	53.965	23.888	2.626	0.007714

The relative permittivity (ε_r_) and conductivity (σ) are given for the solution and cuvette as a function of frequency.

### CPE assay

After RF, sham, or heat exposure, each BCoV sample was diluted from 10^0^–10^−4^ in SFM and added to HRT-18G cells (plated at 15,000 cells per well 24 h the day before). Prior to infection, cell monolayers were rinsed twice with SFM and then inoculated with 20 µL of BCoV samples. Cells were incubated for 1 h (37 °C, 5% CO_2_, 95% RH) and 80 µL of EMEM containing 2% FBS was added to each well. Virus inoculated cells were incubated for 6 days to allow infection to occur. On Day 6 post-inoculation (D6 pi), three independent researchers evaluated each well microscopically to determine if a CPE was present. To prevent bias, researchers were blind to the identity of the samples. After evaluation, the titer of infectious virus was quantified by calculating the TCID_50_/mL for each condition using the Reed-Muench equation^22^. To compare results between multiple experiments (where definite TCID_50_/mL values may differ), we converted the TCID_50_/mL values to % Inactivation (ratio), as follows:

1. $$\%\mathrm{ \,Infectivity}\,=\,\frac{\frac{\mathrm{TCID}50}{\mathrm{mL}}\mathrm{\, exposed\, }(\mathrm{CPE})}{ \frac{\mathrm{TCID}50}{\mathrm{mL }}\mathrm{\, sham \,}\left(\mathrm{CPE}\right)}\mathrm{\times }100,$$
2. $$\% \mathrm{\, Inactivation\, }\left(\mathrm{Ratio}\right)\,=\,100 -\mathrm{\%\, Infectivity}.$$

### Statistics

All experiments were repeated a minimum of three separate times (with three technical replicates per experiment) and the high and low values were subtracted from each condition to eliminate outliers. Data are reported as mean values with error bars representing the standard error of the mean (± S.E.M.). Pairwise comparisons of virus titers (TCID_50_/mL) or % inactivation for each RF or heat exposure were compared to sham using one-way analysis of variance with Dunnett’s multiple comparisons test and Student’s t-test. The criterion for significance was set at a p-value < 0.05 for type I error.

## Results

### Heat inactivation of BCoV

To establish a baseline efficacy of viral inactivation, we performed control exposures using heat to inactivate BCoV. Figure 2 shows % inactivation after heating of BCoV in suspension (B,C) or dried on glass slides (D,E) as measured by CPE analysis. Heat control experiments were run in triplicate and CPE was evaluated at D6 pi. Results show complete (i.e., 100%) inactivation of BCoV in solution after 15 min of exposure to 60 °C temperature (Figs. 2B–C). In comparison, complete neutralization of BCoV deposited on 25 × 75mm glass slides was not observed until application of 80 °C for 15 min (Figs. 2D–E).

### Cuvette RF modeling and exposure dosimetry

For the present study, exposures were conducted in a system that was a replication of a system described by Yang et al. previously^12^. Figure 3A depicts the setup for the cuvette exposure conditions. For each of eight frequencies utilized in the cuvette/solution exposure study, we observed relatively uniform EM fields across the spatial extent of the cuvette and solution within the simulated exposure geometry. A representation in Fig. 3B depicts the two-dimensional power density distribution relative to the cuvette/solution for the 6 GHz condition. Figure 3C summarizes all power densities for the cuvette and solution. Table 1 lists the mean ± standard deviation (SD) incident power densities, where the values are determined by sampling over the surface area of the cuvette and solution in the free-space plane at 5 cm from the horn aperture, centered on boresight (Table 2).

**Table 2:** The incident power density (iPD) of the spatial extent representing the surface area of the cuvette and solution in the free-space plane.

Frequency (GHz)	iPD (Cuvette) (W/m^2^)	iPD (Solution) (W/m^2^)
6.0	1110 ± 145	1040 ± 103
7.0	1090 ± 147	1020 ± 102
8.0	897 ± 61.0	880 ± 44.0
8.4	899 ± 67.0	897 ± 52.6
9.0	843 ± 41.5	849 ± 32.9
10.0	787 ± 113	860 ± 80.5
11.0	798 ± 183	910 ± 121
12.0	734 ± 206	871 ± 133

Data are shown as mean ± SD, and each sample was placed 5 cm away from the horn aperture centered on boresight.

With the cuvettes filled with 1 mL of solution positioned in front of the 6.3 W horn, the voxel SAR values for the solution were calculated according to Eq. (1). A candle plot of the SAR values at each frequency are given in Fig. 3D, where the limits of the whiskers are set to the 1 and 99%-tile SAR voxel values. The range of SAR values increases as a function of frequency. The increase is attributed to the decrease in wavelength and penetration depth, where more energy deposition occurs towards the surface of the cuvettes.

Additionally, given the propensity for RF exposures to generate heat, we evaluated the thermal gradient during exposures. Figure 3E depicts the thermal gradient curves, as measured using a fiber-optic temperature sensor (Opsens, Inc., Quebec, Canada) placed within the cuvette solution during exposure. The measurements were taken in two cuvettes simultaneously (averaged together) across two complete trails starting at room temperature. Results show the change in temperature (ΔT in °C) was between 9 and 19 °C, depending on the exposure frequency (Fig. 3E), which is in line with previous studies^12^. The maximum temperature achieved in the experiment was 39 °C, well below the threshold required to neutralize coronavirus at clinically relevant levels (Fig. 2)^23,24^.

### Cuvette RF exposures

**Figure 4:**
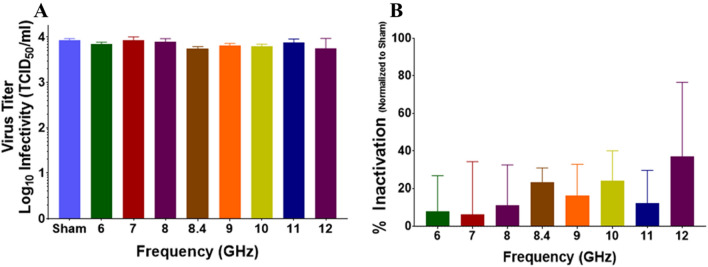
Infectivity of BCoV in solution after exposure to RF. Viral infectivity was assessed by CPE evaluation. Viral titers were determined upon titration on HRT-18G cells, as described in the methods, and evaluated at 6 days post inoculation. (**A**) Virus titer (TCID_50_/mL) following exposure to specific GHz frequencies with 6.3 W applied power for 15 min. (**B**) % Inactivation (i.e., data normalized to non-exposed virus sham) following exposure to specific GHz frequencies with 6.3 W applied power for 15 min durations. Data are expressed as mean values ± S.E.M. of at least three independent experiments (n = 3).

Our first set of experiments studied BCoV in solution. For each independent RF exposure, we performed a sham (mock) exposure where the sample was placed in front of the horn in the experimental configuration, but the RF source remained off. This condition served as a negative control. As described in the methods section, we exposed solutions of BCoV in plastic cuvettes to RF using a system that was tunable to specific frequencies between 6 and 12 GHz. Exposures were performed in triplicate at each frequency and, since two cuvettes were exposed at one time, we achieved an n = 6 for each experimental condition. Figure 4 depicts results for cuvette exposures. Results show no statistically significant difference in virus titer (TCID_50_/mL) or % Infectivity in any of the exposed conditions compared to sham (Fig. 4A and B, respectively). It should be noted that conversion of the data from TDIC_50_ to % infectivity magnifies error of measurement as observed in the 12 GHz exposures. While having relatively high variability it should be noted that our observation of viral infectivity on the linear scale are not considered clinically relevant. We chose to present data in this form so that authors could make direct comparison to previous data^12^ and also small change in viral infectivity against sham exposures could be clearly seen. As the method of viral assessment was CPE measurements of changes under log scale will contain significant error.

**Figure 5:**
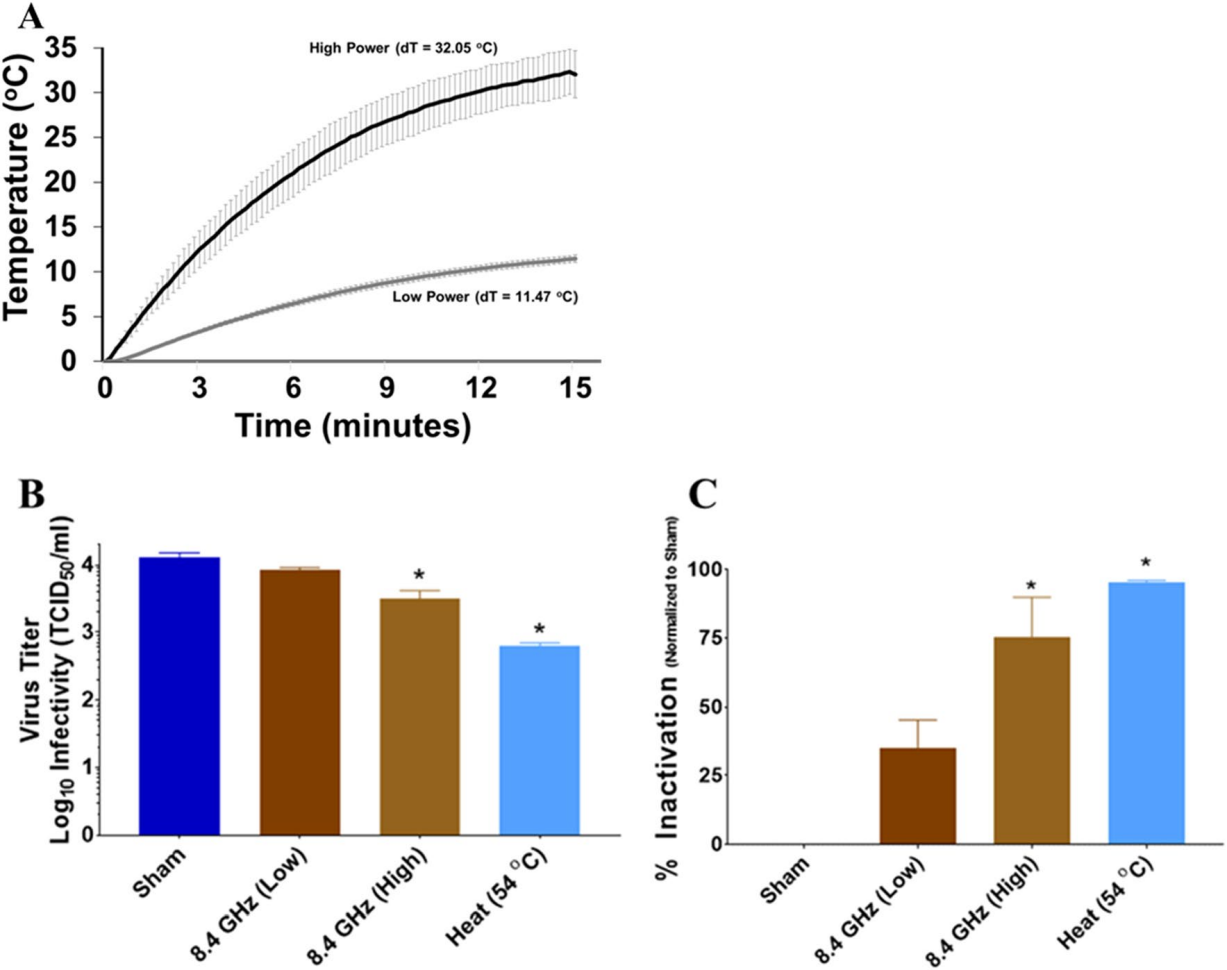
Infectivity of BCoV in solution after exposure to RF with high-applied power. Viral infectivity was assessed by CPE evaluation. Viral titers were determined upon titration on HRT-18G cells, as described in the methods, and evaluated at 6 days post inoculation. (**A**) Temperature profile from the cuvette exposures, measured by Opsens during the 15-min duration at higher applied power. (**B**) Virus titer (TCID_50_/mL) following exposure to 8.4 GHz frequencies with low (6.3 W) or high (30.0 W) applied power for 15 min durations compared to a matched heating. (**C**) % Inactivation (i.e. data normalized to non-exposed virus sham) following exposure to specific GHz frequencies with low (6.3 W) or high (30.0 W) applied power for 15 min durations compared to a matched heating. Data are expressed as mean values ± S.E.M. of at least three independent experiments (n = 3). Statistically significant differences are noted by an asterisk, which represents p-value < 0.05.

Since the exposure conditions utilized in the exact replication of the Yang et al.’s experimental design did not significantly inactivate BCoV, we attempted to drive inactivation of BCoV in solution by increasing applied power^12^. To accomplish this, we increased the applied power to 30 W (max power generated by our system) at 8.4 GHz, which increased the temperature of the BCoV solution to 54 °C at the end of the exposure (Fig. 5A). Compared to low power (6.3 W applied) exposures, which did not produce significant inactivation of BCoV, exposure to high power (30 W applied) produced heat and a significant, 75% inactivation of the BCoV in solution (Figs. 5B and C).

### Coupon RF modeling and exposure dosimetry

**Figure 6:**
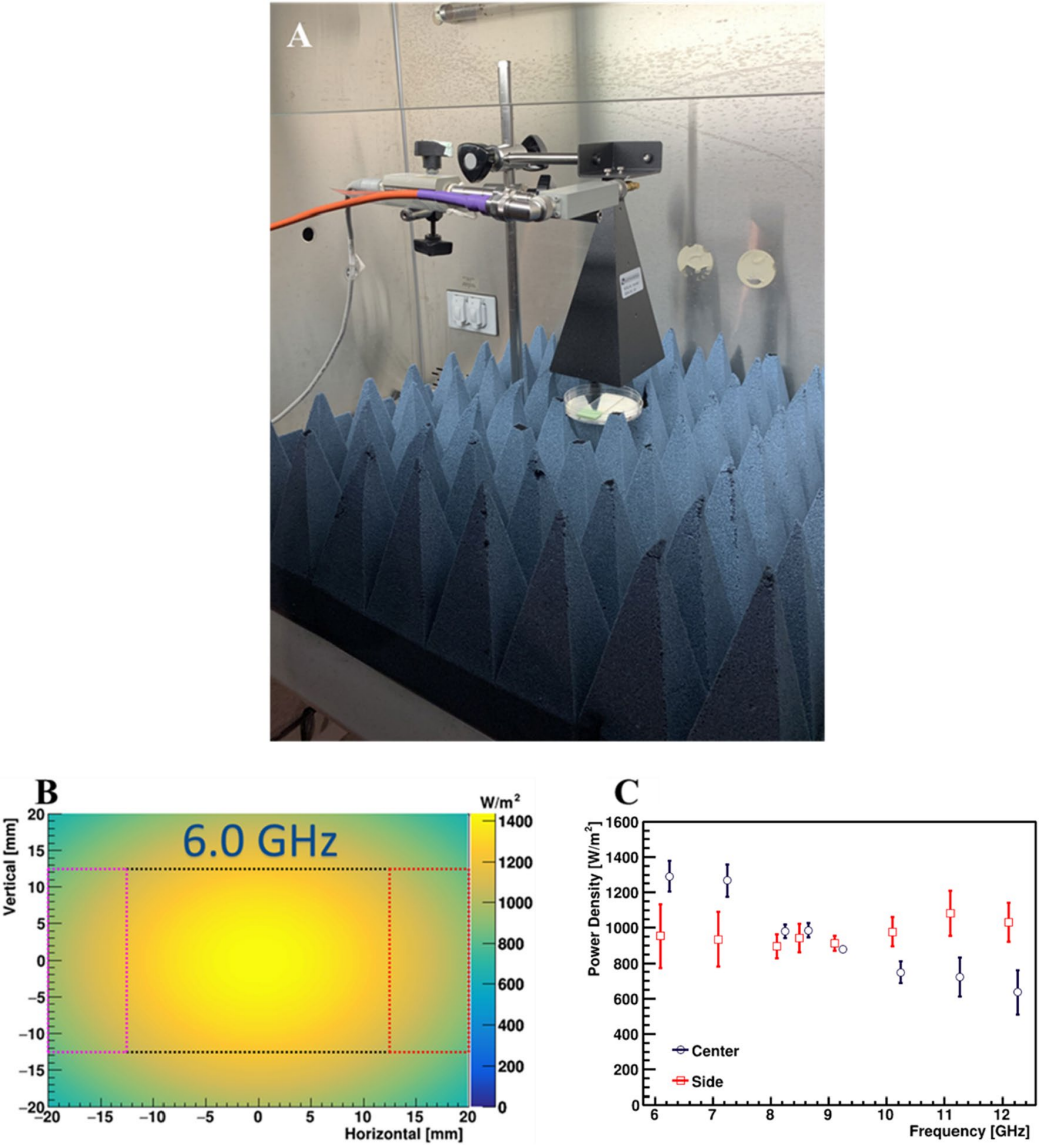
Characterization of the coupon exposure system. (**A**) Photograph displays the experimental setup for exposure of BCoV deposited on a glass slide. (**B**) Representative numerical simulation of the exposure area using XFdtd® simulation software. The squares in the middle of the simulation show the position of the dried BCoV sample within the field. (**C**) The power density was quantified based on the XFdtd® simulation for each exposure and are reported as mean ± standard deviation for each frequency.

The RF exposure system was reconfigured for surface/coupon exposure as described by Yang et al. (Fig. 6A)^12^. For each of the eight frequencies utilized in the coupon/surface exposure study, we observed uniform EM fields across the spatial extent of the glass slide within the simulated exposure geometry. A representation in Fig. 6B shows the two-dimensional power density distribution relative to the coupon/surface for the 6 GHz condition. Figure 6C summarizes all power densities for the cuvette and solution. Table 3 lists the mean ± SD incident power densities, where values were determined by sampling over the surface area of the coupon in the free-space plane at 5 cm from the horn aperture.

**Table 3:** The incident power density mean ± SD of the spatial extent representing the surface area of the exposed coupon in the free-space plane 5 cm away from the horn aperture centered on boresight.

Frequency (GHz)	Power density (W/m^2^)
6.0	1200 ± 192
7.0	1180 ± 186
8.0	956 ± 60.0
8.4	973 ± 57.8
9.0	888 ± 32.4
10.0	811 ± 123
11.0	821 ± 197
12.0	744 ± 215

### Coupon RF exposures

**Figure 7:**
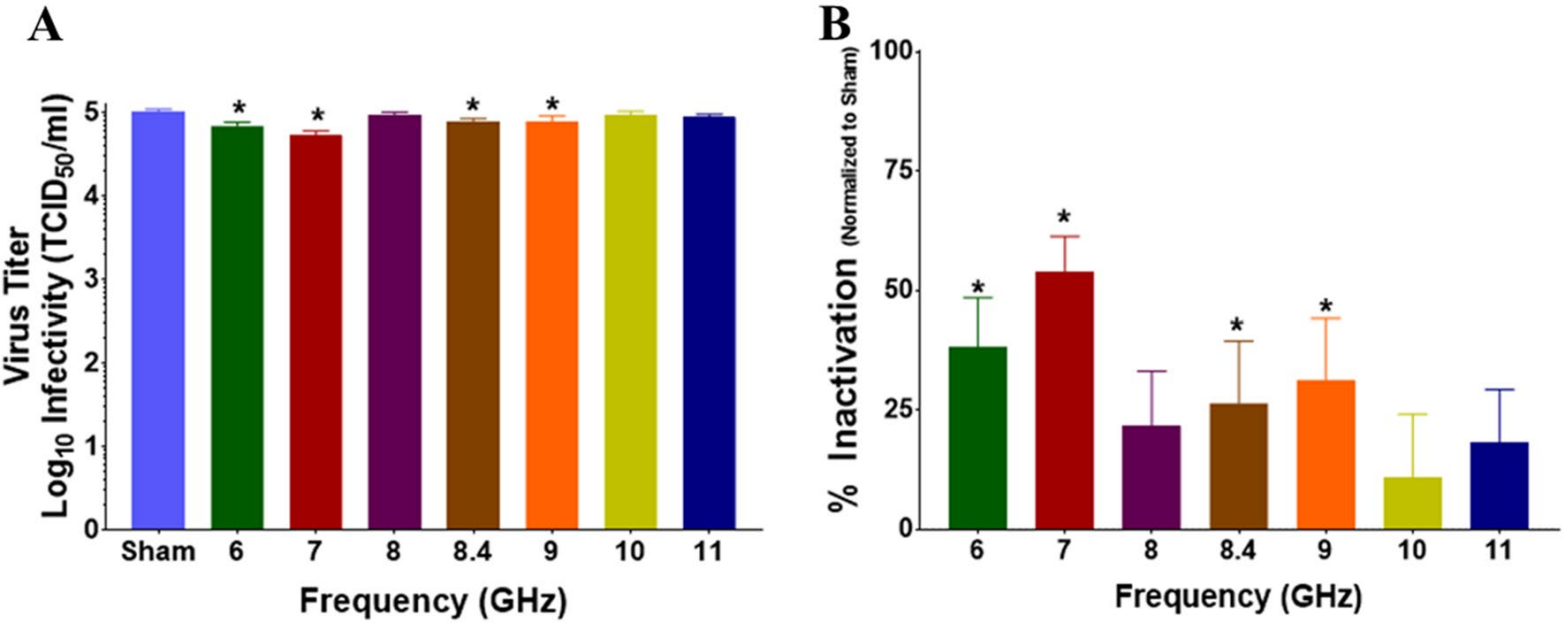
Infectivity of BCoV on a glass surface after exposure to RF. Viral infectivity was assessed by CPE evaluation. Viral titers were determined upon titration on HRT-18G cells, as described in the methods, and evaluated at 6 days post inoculation. (**A**) Virus titer (TCID_50_/mL) following exposure to specific GHz frequencies with 6.3 W applied power for 15 min durations. (**B**) % Inactivation (i.e. data normalized to non-exposed virus sham) following exposure to specific GHz frequencies with 6.3 W applied power for 15-min durations. Data are expressed as mean values ± S.E.M. of at least three independent experiments (n = 3). Statistically significant differences are noted by an asterisk, which represents p-value < 0.05.

For each RF exposure, performed independently, we performed an equivalent sham exposure where the sample was placed beneath the horn in the experimental configuration, but the RF signal turned off. As described in the methods section, we exposed BCoV samples deposited and dried on glass slides to RF using a system that was tunable to specific frequencies between 6 and 11 GHz. Exposures were performed in triplicate at each frequency. Figure 7 depicts results for these exposures. We observed a significant reduction in virus titer (Fig. 7A) and a corresponding significant increase in % inactivation (Fig. 7B) following exposure to 6, 7, 8.4, and 9 GHz. A maximum % inactivation of 53.9 ± 7.4% was achieved after exposure to 7 GHz (Fig. 7B). Importantly, while this is a statistically significant result, it does not meet clinical significance for neutralization, which would require > 3 log_10_ reduction in virus titer or > 99% inactivation.

**Figure 8:**
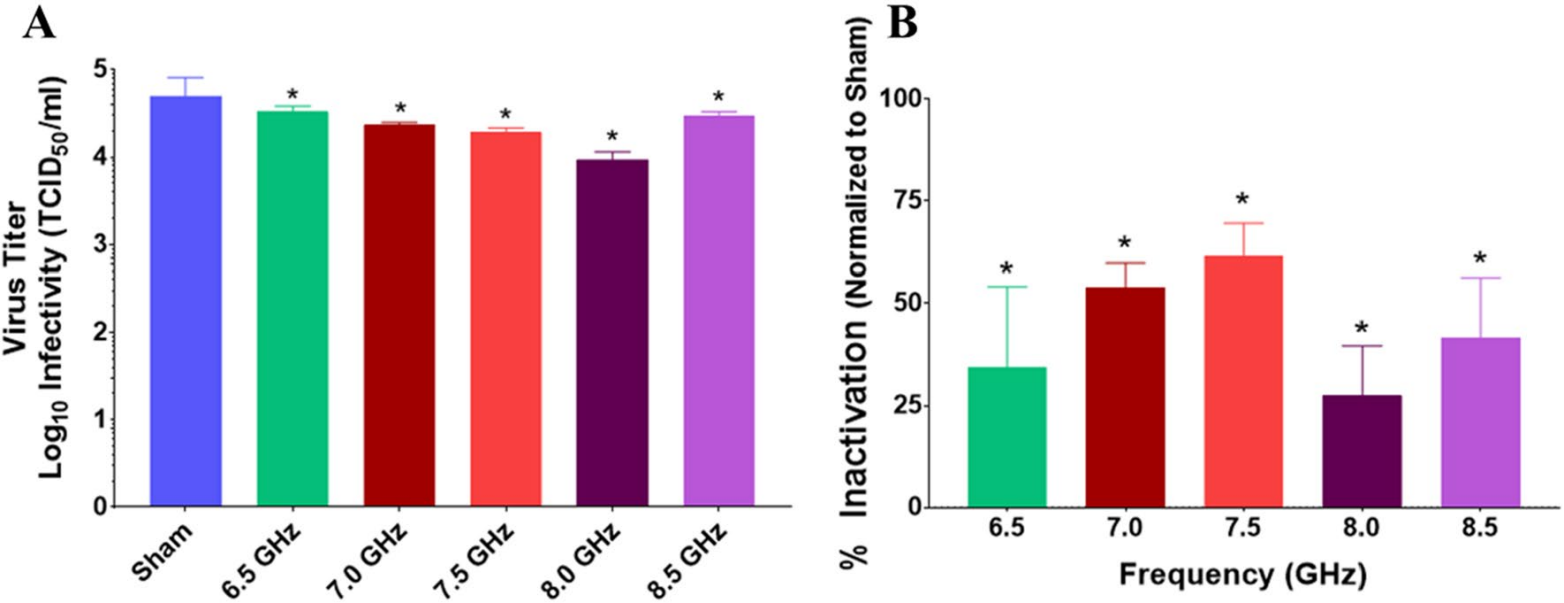
Infectivity of BCoV on a glass surface after exposure to RF: Expanded Frequency Range. BCoV was exposed to specified RF fields between 6.0 and 9.0 GHz in half step increments. Viral infectivity was assessed by CPE evaluation. Viral titers were determined upon titration on HRT-18G cells, as described in the methods, and evaluated at 6 days post inoculation. (**A**) Virus titer (TCID_50_/mL) following exposure to specific GHz frequencies with 6.3 W applied power for 15-min durations. (**B**) % Inactivation (i.e., data normalized to non-exposed virus sham) following exposure to specific GHz frequencies with 6.3 W applied power for 15-min durations. Data are expressed as mean values ± S.E.M. of at least three independent experiments (n = 3). Statistically significant differences are noted by an asterisk, which represents p-value < 0.05.

Since initial experiments did not achieve clinically relevant levels of BCoV neutralization (i.e., > 99% inactivation), we refined the frequency sampling interval to investigate whether a resonant peak for inactivation could be found in the 6.5–8.5 GHz range. Therefore, the experiment was repeated in half GHz frequency steps (6.5, 7, 7.5, 8, and 8.5 GHz). Results show a significant reduction in virus titer (Fig. 8A) and a corresponding significant increase in % Inactivation (Fig. 8B) of BCoV at all frequencies tested. However, none of the conditions reached the criteria (> 3 log_10_/99% inactivation) to be considered clinically significant.

**Figure 9:**
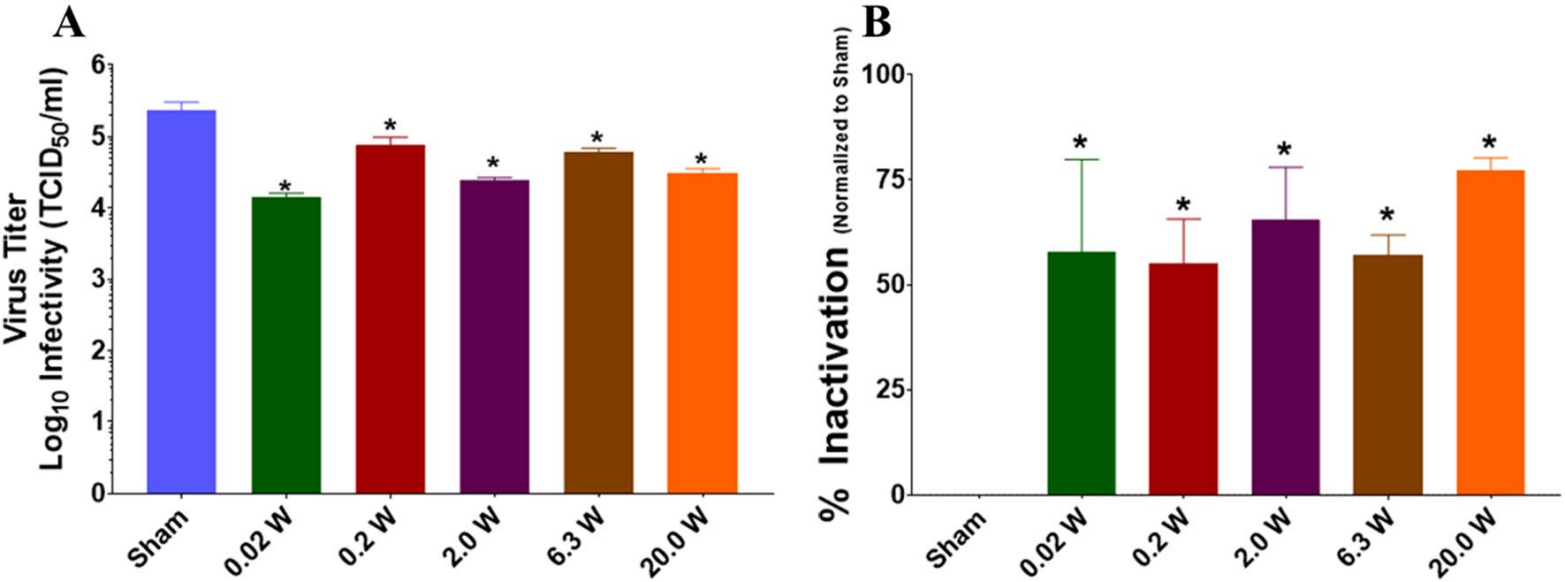
Infectivity of BCoV on a glass surface after RF exposure for variable applied power. Viral infectivity was assessed by CPE evaluation. Viral titers were determined upon titration on HRT-18G cells, as described in the methods, and evaluated at 6 days post inoculation. (**A**) Virus titer (TCID_50_/mL) following exposure to specific GHz frequencies with 6.3 W applied power for 15 min durations. (**B**) % Inactivation (i.e., data normalized to non-exposed virus sham) following exposure to 7.0 GHz RF with variable applied power (0, 0.02, 2.0, 6.3, or 20.0 W) for 15 min durations. Data are expressed as mean values ± S.E.M. of at least three independent experiments (n = 3). Statistically significant differences are noted by an asterisk, which represents p-value < 0.05.

Next, we examined the relationship between applied power and inactivation of BCoV. For this, we exposed BCoV on glass slides to a range of applied powers (0.02, 0.2, 2.0, 6.3, or 20.0 W) at 7 GHz (our most effective frequency) and evaluated virus titer/% inactivation. Results show a significant reduction in virus titer (Fig. 9A) and a corresponding significant increase in % inactivation (Fig. 9B) of BCoV at all powers tested. Importantly, while increasing the power to the maximum applied level (20.0 W) significantly increased the reduction in virus titer compared to 6.3 W applied, we could still only achieve 77.4 ± 2.9% inactivation, which does not meet the criteria for clinical significance.

**Figure 10:**
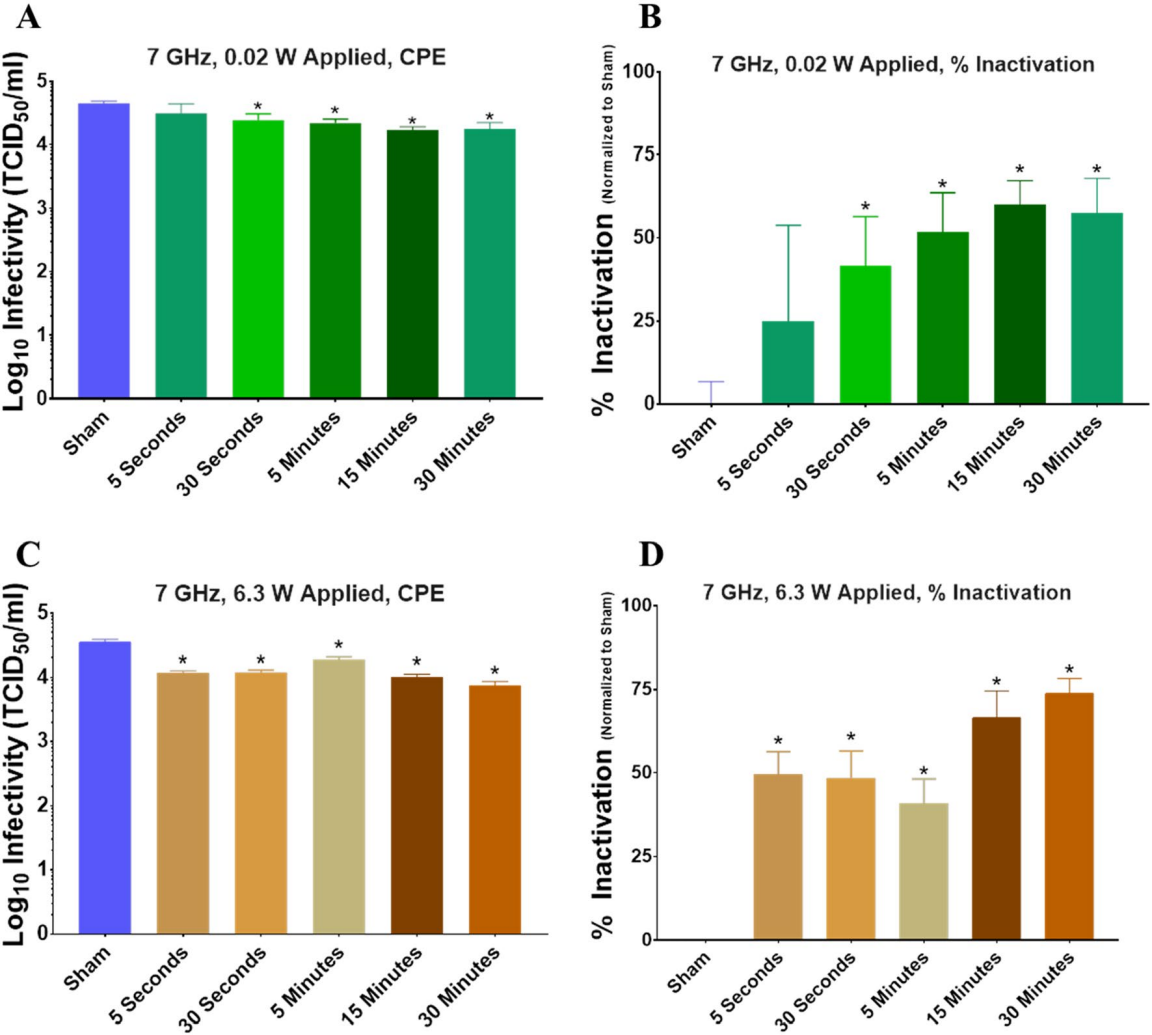
Infectivity of BCoV on glass surfaces after RF exposure for variable time. Viral infectivity was assessed by CPE evaluation. Viral titers were determined upon titration on HRT-18G cells, as described in the methods, and evaluated at 6 days post inoculation. (**A,B**) Virus titer (TCID_50_/mL) or % Inactivation (i.e. data normalized to non-exposed virus sham) following exposure to 7.0 GHz RF with 0.02 W applied power for variable duration (5 s, 30 s, 5 min, 15 min, or 30 min). (**C,D**) Virus titer (TCID_50_/mL) or % Inactivation (i.e. data normalized to non-exposed virus sham) following exposure to 7.0 GHz RF with 6.3 W applied power for variable duration (5 s, 30 s, 5 min, 15 min, or 30 min). Data are expressed as mean values ± S.E.M. of at least three independent experiments (n = 3). Statistically significant differences are noted by an asterisk, which represents p-value < 0.05.

As a final investigation of the effects of RF on the neutralization of BCoV, we reduced and/or extended exposure time to see if changing the duration of exposure would change reduction in infectivity. Exposures were conducted at 7 GHz at either 0.02 W (Fig. 10A–B) or 6.3 W (Fig. 10C–D) applied power for a range of exposure times (5 s, 30 s, 5 min, 15 min, or 30 min). Results showed a significant reduction in virus titer (Fig. 10A) and a corresponding significant increase in % inactivation (Fig. 10B) at exposure durations of longer than 30 s with 0.02 W applied power. Similarly, we observed significant reduction in virus titer (Fig. 10C) and a corresponding significant increase in % inactivation (Fig. 10D) at all exposure durations with 6.3 W applied power. In summary, prolonging the exposure did not significantly enhance RF-induced neutralization of BCoV.

## Discussion

Previous studies showed that viruses (e.g., influenza) exposed to RF frequencies between 6–12 GHz range were 100% inactivated, which was hypothesized to be due to induced electromechanical forces disrupting the viral membrane^12^. To investigate this effect in the context of coronavirus, we exposed BCoV in solution and dried on a surface using the same RF exposure system and geometry^12^. Our dosimetry shows we were able to match the power densities reported by Yang et al. (880 ± 44.0 W/m^2^ versus 810 W/m^2^ at 8 GHz)^12^. Additionally, we validated that our conditions did not produce sufficient heating to neutralize BCoV at clinically relevant levels (Figs. 3D–F). Therefore, any clinically relevant neutralization observed could not be attributed solely to bulk heating of the sample and must include other mechanism(s) of interaction such as SRET^12^ or direct effects on the spike proteins^25,26^.

Given similarities between the molecular structure of influenza and coronavirus (Fig. 1), we hypothesized that we would see similar disruption of the virion resulting in its inactivation. However, although we observed some statistically significant changes in viral inactivation in the 6–9 GHz frequency range on exposed a surface (i.e., glass slide), all of our results remained well under 1 log_10_ kill (< 90%), which translated to roughly 30–60% inactivation, depending on the exposure conditions. With respect to antiviral technologies, and to put these results into a broader context, true antiviral technologies must achieve at least 3 log_10_ (> 99.9%) reduction in infectivity to be considered reasonably effective. Therefore, although we achieved neutralization of BCoV, levels of inactivation would not be considered biologically relevant in the context of antiviral resolutions. Specifically, under the conditions utilized within the present study, the maximum effect achieved was a 77.4 ± 2.9% inactivation, which did not meet the criteria of clinical significance. Notably, even when we significantly increased the RF dose by varying the exposure duration or power level, we did not find greater than 77% neutralization of the BCoV sample (Figs. 9 and 10). Since a significant number of exposure conditions produced viral inactivation in the 50–70% range, we theorize that the heterogeneity of the BCoV population (e.g., difference in shape or size) might have resulted in a resistance to the effects of RF when exposed at a single frequency. Future studies will explore this potential RF-resistant population in more detail to determine if there is a structural or biological feature of the virus that leads to its survivability in RF fields.

Furthermore, while we did not observe a complete (i.e. 100%) reduction in virus inactivation as observed in the Yang et al.’s study^12^, there are a few differences between the two investigations that make direct comparison difficult. Most importantly, we used a different virus (BCoV) to better match the shape and size of SARS-CoV-2 (Fig. 1)^16–19^. Previous approaches to studying coronavirus neutralization focused primarily on surrogate agents including influenza A or bacteriophage MS2, due to difficulties associated with culturing SARS-CoV-2 and the need for biosafety level 3 (BSL3) containment to handle the virus. While these studies were informative, differences in virus composition between non-enveloped viruses such as MS2 made it difficult to conclude that similar effects would occur when treating coronavirus. Therefore, we selected BCoV as a surrogate for SARS-CoV-2 based upon similarities in size and composition between the two beta-coronaviruses (Fig. 1) as well as its biosafety level (BSL2). Our selection of BCoV provided greater reliability in translating our experimental results to RF technologies relevant to coronavirus neutralization. It is unclear if the interaction of the RF field varies based on virus species. However, given the similarities in size, shape, and biological composition (Fig. 1), it is likely that if an electromechanical mechanism is responsible for the neutralization of influenza A, it would be applicable to other similarly structured viruses, including coronavirus. However, since we do reach 60–70% inactivation of BCoV following a similar exposure paradigm, it is possible that the electromechanical mechanism(s) originally theorized by Yang et al. could be applicable to a sub-population of our sample^12^. Given that acoustic vibrations are heavily dependent on the size and mass of the virion, we speculate that difference observed between our exposures and those previously reported may be in the virion species itself. Given the larger size of our virus, we would expect a shift in frequency to a lower range. Also, the mass of the virus is also different which could impact the resonant frequency substantially^27^.

From this study, we cannot prove that SRET is occurring within BCoV upon exposure to RF fields. However, we did find that the overall effect appeared to be much less defined than in previous publications, with a “specific” frequency region being much less convincing. This would suggest that the resonant frequency of the virus is either very broad or that another mechanism can account for the loss of viral infectivity during RF exposure. Our measure of viral infectivity could arise from multiple disruption mechanisms like viral DNA damage, inhibition of spike protein binding to the host cell, or whole virus destruction by heat or SRET. Recent models of DNA and RNA from virus show resonant frequencies of 180 GHz required to cause damage, notably higher than what was used in this study^28^. Very recent work has modeled SARS-CoV-2 virus for mechanical resonance and found that 80–120 MHz to be the optimum frequency, well below that being used in this study^13^. Using these two results as endcaps for the resonance argument, we hypothesis that the up to 70% reduction in viral infectivity observed in our studies is not due to DNA damage or whole virus resonance (as reported^12,14^). As evidenced by our data, we saw very little change in infectivity in bulk exposure of virus within the cuvette, which also carried less thermal rise than would be expected to denature the virus directly^24^. However, upon placing the virus on a coupon, to simulate a surface, and allowing it to dry, we observed some changes in viral infectivity. From this, we hypothesize two possible mechanism(s). The first is that water left within the sample (amongst proteins and lipid membranes) is heated by the RF exposure creating a thermal gradient resulting in damage. This hypothesis should be tested through modeling of the nanometer scale thermal environment of the virus during exposure. The second hypothesis is that RF is having a direct impact on spike protein orientation limiting its effectiveness to bind to the host cell^25,26^. This hypothesis could be tested by analyzing changes in spike protein orientation within viral particles via FRET or Raman methods.

In conclusion, we evaluated whether low power, CW, RF exposures could inactivate a surrogate coronavirus in the 6–12 GHz range. We observed a statistically significant reduction in viral inactivation on surface preparations; however, this neutralization does not reach clinical significance (> 99% inactivation). Further, the results of the present study, in combination with a lack of definitive theoretical mechanism from previous work, suggest that large area RF exposure would not limit the viability of virus on surfaces and objects under the conditions tested herein. Therefore, at this time we cannot recommend the use of RF technologies to neutralize coronavirus under the conditions tested within the present study. Future studies will focus on virion-level analysis during and after EM field exposure to study mechanism(s) of action resulting in the observed inhibition. Determination of which physical forces are acting on or within the virion will enable more precise tuning of exposure parameters, which may enable clinically-relevant inhibition levels. Additional validation of this mechanism across other virus species should also be performed, to not only aid in determination of mechanism of interaction, but also validate whether or not such mechanism can be broadly applied as an anti-viral technology.

## Data Availability

The datasets generated during and/or analyzed during the current study are available from the corresponding author on reasonable request.
